# 1826. Comparative Use of Vancomycin versus Ampicillin for Blood Stream Infections Caused by Ampicillin-susceptible *Enterococcus faecalis*

**DOI:** 10.1093/ofid/ofac492.1456

**Published:** 2022-12-15

**Authors:** Michaele Phan, Jacqueline Meredith, Renee Ackley, Alex Ebeid, Kristen Goodrich, Mindy Sampson

**Affiliations:** Atrium Health Carolinas Medical Center, Charlotte, North Carolina; Atrium Health’s Carolinas Medical Center, Charlotte, North Carolina; Atrium Health, Charlotte, North Carolina; High Point University Fred Wilson School of Pharmacy, Germanton, North Carolina; UNC Eshelman School of Pharmacy, Cary, North Carolina; Atrium Health, Charlotte, North Carolina

## Abstract

**Background:**

Available studies assessing the efficacy of beta-lactam antibiotics compared to vancomycin (VAN) in the treatment of Enterococcal blood stream infections (BSI) have varying results and have not assessed the impact of rapid diagnostic testing. We aimed to compare clinical outcomes of ampicillin (AMP) vs. VAN for *E. faecalis* BSI treatment.

**Methods:**

A multisite, retrospective evaluation of adults with *E. faecalis* BSI between 2017 to 2021 was performed. Patients who received at least four days of AMP or VAN as definitive therapy were analyzed; only initial episodes were included. All positive blood cultures underwent multiplex polymerase chain reaction blood culture identification (BCID). Patients with polymicrobial BSI were excluded, or if they received ≥ 50% concomitant use of AMP and VAN. The primary outcome was 30-day all-cause mortality. Secondary objectives included 90-day all-cause mortality, hospital length of stay, incidence of treatment failure, persistent BSI, change from initial definitive therapy, and adverse drug reactions (ADRs). Other microbiology endpoints included time to active treatment, BCID, beta-lactam coverage, definitive therapy, and culture clearance. A sample size of 208 patients was estimated to provide a 15% mortality difference with 80% power.

**Results:**

In total, 123 patients with *E. faecalis* BSI were analyzed (AMP, n = 92; VAN, n = 31). Baseline characteristics were similar among both groups except gender and baseline penicillin allergy (p < 0.001) (Table 1). All-cause 30-day mortality was not significantly different between patients treated with AMP and VAN (10.8% vs. 22.6%, p = 0.130). No difference was found in secondary objectives except time to definitive therapy was longer for the AMP treatment arm (56.7 hours vs. 15.8 hours, p < 0.001) (Table 2). The incidence of ADRs were similar between the two groups (Table 3).

**Figure:**
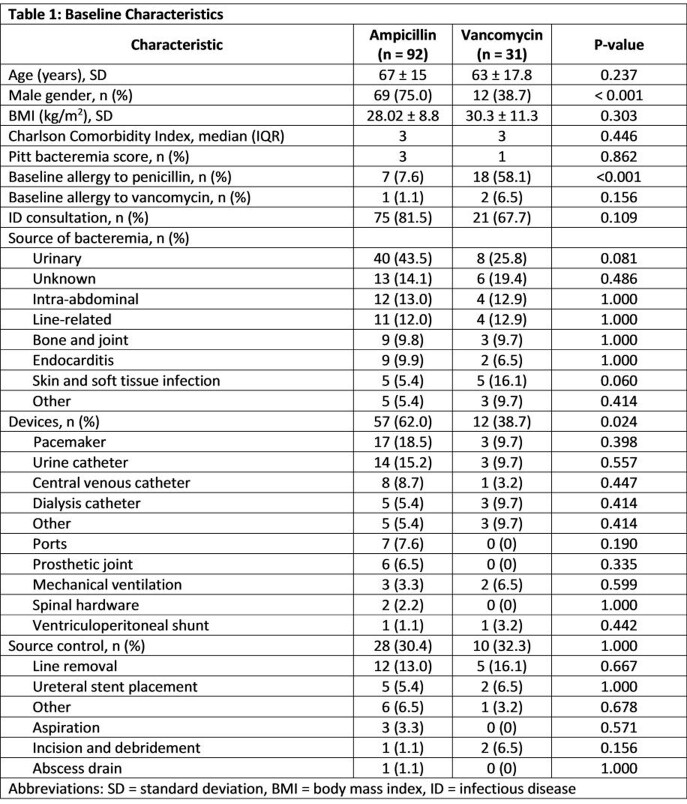

**Figure:**
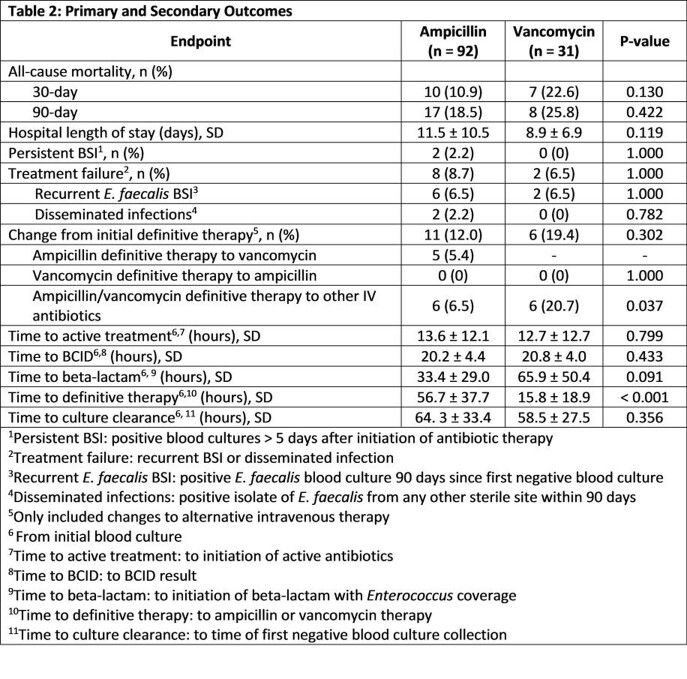

**Figure:**
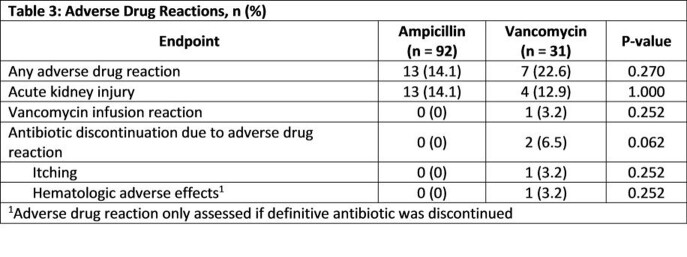

**Conclusion:**

No difference in mortality was observed in the treatment with AMP or VAN for patients with *E. faecalis* BSI, but the study was not sufficiently powered. Despite the use of rapid BCID testing, earlier initiation of AMP was not observed. AMP or VAN may be reasonable options for treatment of *E. faecalis* BSI, but further research is warranted.

**Disclosures:**

**All Authors**: No reported disclosures.

